# Functional Traits of Native Plant Species That Inhibit the Seedling Growth of the Exotic Invader *Solidago canadensis*

**DOI:** 10.3390/plants14172806

**Published:** 2025-09-08

**Authors:** Ruixiang Ma, Jili Liang, Keyi Zuo, Ming Wu, Xiaoqi Ye

**Affiliations:** 1 Research Institute of Subtropical Forestry, Chinese Academy of Forestry, Hangzhou 311400, China; rxma@caf.ac.cn (R.M.); liangjili124@163.com (J.L.); zuokeyi@caf.ac.cn (K.Z.); hangzhoubay@126.com (M.W.); 2 State Key Laboratory of Wetland Conservation and Restoration, Chinese Academy of Forestry, Beijing 100091, China; 3 College of Ecology and Environment, Nanjing Forestry University, Nanjing 210037, China; 4 College of Forestry and Biotechnology, Zhejiang Agriculture and Forestry University, Hangzhou 311300, China

**Keywords:** *S. canadensis*, native plants, functional traits, competition

## Abstract

Rising biological invasions continue to threaten biodiversity conservation worldwide. To protect native ecosystems and biodiversity, improve resilience against invasions, and lower ecological management costs, it is crucial to identify native plant species that can endure the competitive pressures from invasive plants. This greenhouse study examined the competition between *Solidago canadensis* and 32 native plant species to identify key functional traits of these native plant species that influence their competitive effects on and responses to *S. canadensis*. The results indicated that *S. canadensis* seedlings were unable to suppress the growth of most of the native species studied, while most native species could significantly suppress growth of *S. canadensis*, reducing its biomass by 12–92%. The suppression effects by native plants were closely related to their root functional traits. Specifically, annuals with higher root–shoot ratio, specific root lengths, stem biomass, plant height by day 10, and smaller number of root tips showed stronger inhibition of *S. canadensis*. On the other hand, perennials with smaller average root diameter, or greater root biomass and plant heights by day 60, were also more inhibitory towards *S. canadensis*. This study concluded that the competitive effect of seedlings of *S. canadensis* have weaker competitive impacts compared to most the studied native plants. Root traits are essential in the competition between native plants and *S. canadensis*, potentially aiding in the identification of native plant species with high resistance to invasion.

## 1. Introduction

As globalization accelerates process, biological invasions have become a global environmental issue [1,2,3,4]. Invasive plants, with their strong competitive abilities, often form monocultural communities that restrict the living space of native plants, disrupt biodiversity [5,6], alter soil properties [7,8] and modify ecosystem functions [9]. This poses a significant threat to native ecosystems. Therefore, it is crucial to prevent further invasions, eliminate invasive plants, and restore native vegetation. The successful invasion of plants is determined by the ability to overcome local environmental conditions, including both biotic and abiotic factors [10,11]. In order to colonize and reproduce in a new environment, invasive plants must adapt to abiotic environmental conditions (e.g., climate, soil, moisture, light, etc.) [11]. In addition, the interactions between native organisms and invasive species have a significant impact on successful invasion. Invaders must overcome native community resistance mechanisms, including interspecific competition [12], consumer predation pressure [13], and pathogen attack [14]. Nevertheless, anthropogenic manipulation of abiotic filters to prevent invasive species colonization is both economically prohibitive and ecologically unsustainable [15]. Moreover, biological control through natural enemy introduction carries significant unpredictability risks [16]. Therefore, utilizing native plants to compete with invasive plants to defend against plant invasions is more feasible. Currently, besides traditional physical and chemical methods, replacing invasive plants with native plants and developing native vegetations that can resist invasion are essential parts of the integrated management of invasion [17,18]. Therefore, identifying native plant species that are highly resistant to competition or have high abilities to suppress invasive plant species is crucial.

Competitive ability is one of the major components of biological resistance to invasions [19]. High competitive abilities of native plant species may facilitate replacing invasive plants. The strength of plant competitive ability can be assessed by competitive effects (i.e., the ability to inhibit the growth of other species) and competitive responses (i.e., the ability to resist being inhibited by other plants) [20,21,22,23]. Competitive effect and response abilities are often closely related, and plants with strong competitive effect and well response perform have advantage in competition [24,25]. The competitive ability is closely associated with resource utilization efficiency [26]. For example, plants with larger leaf areas and greater plant heights are more advantageous in competing for light resources [27], and a larger proportion of root systems is more conducive to competing for underground resources [28,29,30]. Competitive effect is often related to plant functional traits [31], but competition responses are not significantly correlated with their own functional traits [25], suggesting that the functional traits driving variation in competition responses are not as well defined than competition effects [32]. Mechanisms by which plant functional traits explain competitive interactions between native and invasive species have received increasing attention [33,34]. A multitude of functional traits are associated with plant invasiveness [35,36]. A meta-analysis reported the correlations between functional traits and invasiveness, but the analysis of functional traits did not include root functional traits [35]. Studies on the correlation between functional traits and invasion resistance have focused primarily on above-ground functional traits [37,38]. A study showed that aboveground functional traits could not definitively explain invasiveness and resistance to invasion [39], suggesting the potential roles of belowgound traits. Dawson proposed a new hypothesis that altered root traits can enhance alien plant growth performance and competitive ability [40], and a recent study have demonstrated that invasive plants have significantly different root traits compared to native plants [41]. However, the effects of root traits on the outcome of competition between invasive and native species have not been clearly explained [42,43], perhaps due to the difficulty of obtaining data on root traits and the interference of destructive sampling with experimental results [44], root traits have often been neglected in most studies.

*Solidago canadensis* is native to the temperate regions of North America. Due to its high reproductive and expansion abilities [45], *S. canadensis* has invaded Europe, Asia, and Oceania [46,47]. Studies have found that the invasion of *S. canadensis* has caused changes to the soil properties and microorganisms of the local ecosystem [48] and species diversity [49,50]. Studies on prevention and control of *S. canadensis* have been conducted, however, with limited efficiency [51,52,53]. Using suitable native plants to replace invader can avoid the ecological damage caused by herbicides and physical control, thus contributing to ecosystem health, biodiversity conservation, and sustainable economic and social development. Many studies have been conducted to explain the possible mechanisms of invasiveness of *S. canadensis*, including smaller and lighter seeds for easier dispersal and faster seed germination, as well as the development of extensive underground rhizomes that enhance clonal propagation and the release of allelopathic compounds that negatively affect neighbors [54,55,56]. In addition, larger root biomass is more favorable for *S. canadensis* to compete for underground resources [57], suggesting that roots play an important role in the resource competition process of *S. canadensis*. But the key functional traits for native plant resistance to *S. canadensis* invasiveness remain unclear, making it difficult to screen suitable native plants to replace *S. canadensis*. Based on the limitations of above research, this study aims to elucidate native plants functional traits, that are closely associated with their competitive effects on and responses to *S. canadensis*. We predict that the root traits of native plants play important roles in suppressing growth.

## 2. Results

### 2.1. Changes in Biomass of Native Plants and S. canadensis Before and After Competition

**Figure 1:**
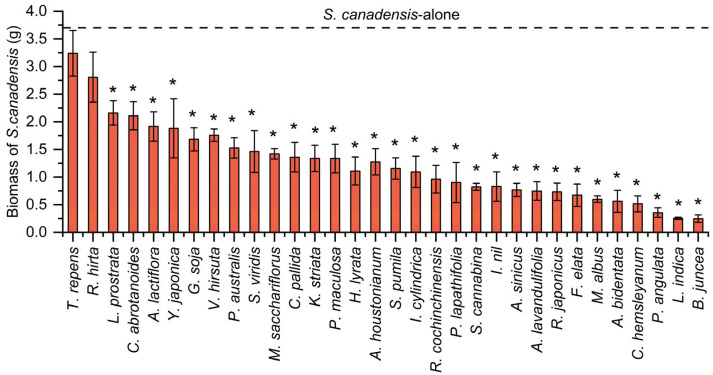
Biomass of *S. canadensis* exposed to competition from different native plants when grown 70 days. The dashed line indicates the mean biomass of *S. canadensis* when grown alone, bars are mean values of *S. canadensis* biomass in competition with different native plants (±s.e., *n* = 5). * indicates statistically significant difference (*p* < 0.05) in biomass between *S. canadensis* grown alone and after competition with native plants.

**Figure 2:**
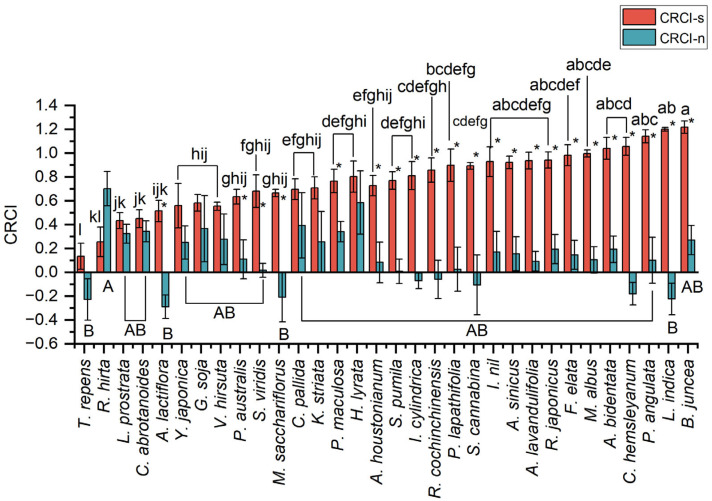
CRCI of *S. canadensis* and native plants. Lowercase letters on the error bars represent significant difference between the CRCI of *S. canadensis* mixed with different native plants, uppercase letters represent significant difference between the CRCI of native plants mixed with *S. canadensis*, and * represents the significant difference between the CRCIs of native plants and *S. canadensis* when they are mixed (*p* < 0.05). Bars are mean values (±s.e., *n* = 5).

**Figure 3:**
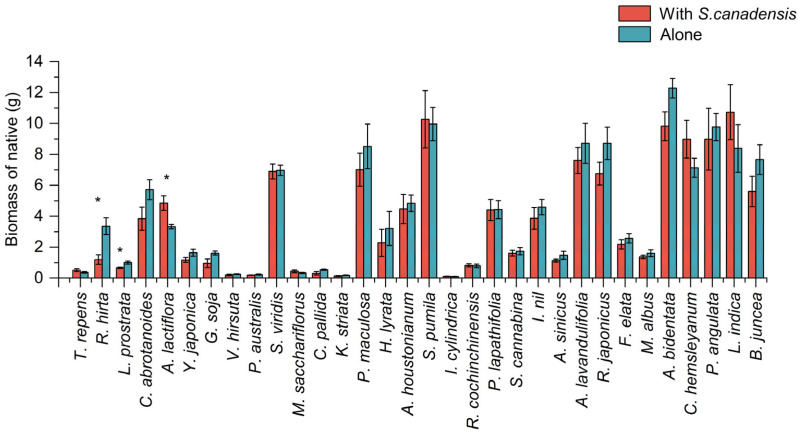
Biomass of native plants grown for 70 days under both competition (with *S. canadensis*) and control treatments. * indicates significant difference (* *p* < 0.05) in biomass between native plants grown alone and after competition with *S. canadensis*. Bars are mean values (±s.e., *n* = 5).

The biomass of *S. canadensis* was lower than that of those planted alone after competition with different native plants (Figure 1), and the biomass reduction in *S. canadensis* ranged from 12–92% (Table S1). *Trifolium repens* and *Rudbeckia hirta* did not significantly inhibit the growth of *S. canadensis* (df = 32, *F* = 12.377, *p* < 0.05). The CRCI for *S. canadensis* varied among the competing native species (Figure 2), but were all positive, indicating a general inhibiting effects from the native species on growth of *S. canadensis* during the seedling stage (df = 31, *F* = 8.197, *p* < 0.05). Specifically, the most pronounced inhibitory effects on the growth of *S. canadensis* were observed in three annual plants: *Brassica juncea*, *Lactuca indica*, and *Physalis angulata*, followed by the perennial plants *Cynanchum hemsleyanum* and *Achyranthes bidentata*. Biomass of native species except *R. hirta* and *Ludwigia prostrata* was not significantly suppressed by *S. canadensis* (Figure 3) and some native plants exhibit a negative CRCI (Figure 2, df = 31, *p* < 0.05), indicating that during the competition, the growth of these native plants is not only uninhibited but also enhanced, leading to an increase in biomass. The biomass of *A. lactiflora* increased significantly (Figure 3). Among the native plant species, only *R. hirta* has an CRCI greater than that of *S. canadensis*, while the CRCI of the remaining native plants during the competition is lower than that of *S. canadensis*.

### 2.2. Correlation Analysis of CRCI with Different Functional Traits in Native Plants and S. canadensis

The CRCI of *S. canadensis* is significantly correlated with multiple functional traits of native plants (Table 1). Specifically, the CRCI of *S. canadensis* is significantly positively correlated with the leaf area (LA), root length (RL), root surface area (RSA), root diameter (RD), root tips number (RT), biomass, and plant height of annual native plants measured from 30 to 70 days after sowing. Additionally, it is significantly positively correlated with the leaf area (LA), RT, biomass, and plant height from 20 to 70 days after sowing of perennial native plants measured (*p* < 0.05). However, the CRCI of perennial native plants was only significantly associated with SLA of *S. canadensis*, and the CRCI of annual native plants was more relevant to root traits such as RL, RSA, and RT (Table 1, *p* < 0.05).

### 2.3. Stepwise Regression Modeling of CRCI and Functional Traits in Native Plants and S. canadensis

The stepwise regression models with the CRCI of *S. canadensis* had good performance (Table 2). Among the functional traits of annual native plants, RSR, M_stem_, plant height at 10 days (H_10_), SRL, and RT are significantly correlated with the CRCI of *S. canadensis*. These functional traits of annual native plants account for 38% of the variation in the CRCI of *S. canadensis*, specifically, the CRCI of *S. canadensis* is positively correlated with M_stem_, RSR, H_10_, and SRL, but negatively correlated with RT (*p* < 0.05). 38.7% of the variation in the CRCI of *S. canadensis* can be explained by the functional traits of perennial native plants, which include RD, M_root_, and plant height at 60 days (H_60_). Among these variables, root biomass (M_root_) and plant height at 10 days (H_60_) are significantly positively correlated with the CRCI of *S. canadensis*, while RD is significantly negatively correlated with the CRCI of *S. canadensis* (*p* < 0.05). However, the adjusted R^2^ of the CRCI of annual and perennial native plants modeled with the functional traits of *S. canadensis* was 0.056 and 0.062 (*p* < 0.05), indicating that the functional traits of *S. canadensis* can be explained 5.6% and 6.2% of the variation in CRCI of annual and perennial native plants (Table S9).

## 3. Discussion

This study showed that during seedling competition between *S. canadensis* and 32 native plants, most of the native plants significantly inhibited the growth of *S. canadensis*, while *S. canadensis* did not significantly inhibit the native plants. In addition, the ability of native plants to suppress the growth of *S. canadensis* at the seedling stage were strongly influenced by their functional characteristics, the functional traits of *S. canadensis* contributed less to its capacity to suppress native plants.

### 3.1. Variation of Competitive Effects Between S. canadensis and Native Plants

The results indicated that native plant species have stronger competitive ability than *S. canadensis* during seeding stages, which is contrasted to the general findings that invasive alien species tend to have greater competitive abilities than native plants species [58]. Some studies have shown that some invasive species grow slowly in the seedling stage and are less competitive than native species [59,60]. Invasive plants exhibit weaker competitive ability at the seedling stage compared to the adult stage, and the seedling stages of invasion were mostly neglected in other research [26]. The weaker competitive ability of *S. canadensis* at the seedling stage may be due to the smaller seed size and therefore less storage of nutrients [56,61], whereas most of the native plant seeds are larger and have a faster growth rate at the seedling stage (Figure S1). However, among the native plants, *T. repens* and *R. hirta* did not significantly inhibit the growth of *S. canadensis*. This may be due to the small biomass and plant height of these two plants (Tables S1–S3), and plants with smaller biomass and plant height usually have weaker competitive ability [62].

Native plants in this study were able to inhibit the growth of *S. canadensis* at the seedling stage, however, *S. canadensis* often competes successfully in the wild [63]. This suggests that there are other potential functional traits that can have an impact on the outcome of long-term competition between native plants and *S. canadensis*. The hypothesis that extended leaf phenology is a key trait for species invasion has been proposed [64] and studies have further confirmed that invasive plants usually have longer growing seasons than native plants [65,66], such as the invasive plants *Lonicera maackii*, *Lonicera japonica*, *Frangula alnus*, and *Berberis vulgaris*, obtain more resources by extending the growing season [65,67]. *S. canadensis* leaf withered significantly later and new leaf grew significantly earlier than native plants, and *S. canadensis* exhibited a higher leaf photosynthetic capacity in fall and winter [68]. Therefore, the phenological differences between *S. canadensis* and native plants during fall and winter probably play a crucial role in the competitive success of *S. canadensis*. However, some native plants that facing competition from *S. canadensis* can coexist with it by completing their life cycle earlier, thereby avoiding direct competition for light resources and soil nutrients [69]. For example, the three native annual plants (*B. juncea*, *L. indica*, and *P. angulata*) that most effectively inhibited the growth of *S. canadensis* in our study grow rapidly and utilize resources efficiently. This trait enables them to complete their life cycle quickly [70], they can leverage phenological differences to quickly establish a competitive advantage in the early stages of the competition, and ultimately exert strong competitive pressure on *S. canadensis*. Therefore, further attention to the effects of phenology on competition between *S. canadensis* and native plants in field conditions is important for suppressing invader and promoting the recovery of native species.

### 3.2. Differences in Functional Traits Associated with Competitive Effects of Native Plants on S. canadensis

Differences in competitive effects between native species and *S. canadensis* can be explained by changes in functional traits associated with competition. Roots likely play a key role in the competition between invasive and native species, and the allocation of root biomass may be positively correlated with the invasiveness of the species [71]. *S. canadensis* competitive effects showed positive correlations with SLA against perennials and with RT against annuals (Table S9). This reflects that higher RT enhanced resource uptake and suppression, whereas similar growth rates between *S. canadensis* and perennials (Tables S2 and S7) intensified light competition. Nevertheless, low model explanatory power (5.3% annuals; 6.2% perennials) is likely due to the slow seedling growth of *S. canadensis* and limited root biomass during early stages (Tables S1 and S4–S7), which likely diminished inhibition of native plants during competition [62]. However, root traits of native plants were significantly correlated with the competitive effects of native plants, where RSR and SRL of annual native plants were positively correlated with the ability to inhibit *S. canadensis* (Table 1 and Tables S9). RSR and SRL are correlated with belowground resource acquisition, when plants have higher RSR and SRL, the root of plants develop more finely and widely distributed in the soil [44,72,73], which allows plants to explore a larger soil volume to obtain more water and nutrient resources, and ultimately has a strong competitive effect on *S. canadensis*. Different from annual native plants, the ability of perennial native plants to suppress *S. canadensis* was significantly negatively correlated with RD and positively correlated with root biomass. Root diameter correlates positively with plant longevity [74], consequently, perennial plants develop larger root diameters for resource storage and defense [75,76,77,78]. Smaller root diameters and greater root biomass enhance soil resource acquisition efficiency [72]. Thus, perennial native plants with these traits can more effectively suppress *S. canadensis*. This supports our hypothesis that native plant root traits play a key role in competition with *S. canadensis*. Beyond belowground traits, plant height also had a significant effect on the ability to suppress *S. canadensis*. Plants with taller heights have greater access to aboveground resources [79]. Annual native species with greater plant height at 10 d after sowing effectively inhibited *S. canadensis* due to rapid growth. In contrast, perennials grew slower than annuals [80]; consequently, those with greater height at 60 d exerted stronger competitive ability on *S. canadensis*. In conclusion, functional traits conferring stronger suppression of *S. canadensis* encompass both aboveground and belowground attributes. This framework facilitates selecting native plants for invasion control by synergistically integrating root and shoot traits.

### 3.3. Optimizing Invasion Control Through Native Plant Traits Suppressing S. canadensis Growth

Research on using native plants to control invader remains limited. A meta-analysis showed that only one-third of the studies actively seeded natives after initial control methods [81]. Moreover, among these studies, few assessed native plant responses holistically, often focusing solely on aboveground effects [82]. Current control methods for *S. canadensis* demonstrate limited efficiency, while integrated approaches combining biological controls with traditional methods (e.g., chemical herbicides and mowing) can temporarily inhibit the growth of the aboveground parts of *S. canadensis* [51,52,83,84,85], without sustained disturbance, *S. canadensis* will quickly regain its competitive advantage through belowground root growth [84]. This further validates the importance of root traits in competition between native plants and *S. canadensis* [86].

Given that the native plants in this study were sown at the same time as *S. canadensis*, our results enable screening native species to suppress *S. canadensis* invasions in newly colonized areas devoid of vegetation. Ecosystems lacking vegetation cover (e.g., bare soil) are particularly vulnerable to plant invasion [87], as observed in abandoned farmland and disaster-disturbed terrestrial ecosystems [88,89]. In such scenarios, sowing native plants with specific functional traits effectively inhibits seed dispersal and colonization of *S. canadensis*. Competition during the seedling stage is different from that of adult plants. Moreover, the increase in aboveground competition during plant development likely coincides with heightened belowground resources competition [90]. Intense belowground competition may trigger biomass reallocation to shoots to compete for more aboveground resources [91]. Therefore, to achieve stage-targeted control of *S. canadensis*, further studies must identify functional traits that confer suppression of the invader across its ontogenetic stages.

As biotic environmental filters, native plant functional traits directly determine the invasion resistance capacity of native ecosystems [11,92]. When native plants exhibit poor resource acquisition capacity (e.g., lower RSR, SRL, and plant height), resulting in underutilized environmental resources, vacant ecological niches become available for invasive species [93]. Global environmental change enhances environmental fluctuations, thereby creating more opportunities for plant invasions [94]. In resource-rich environments (e.g., agricultural fields and riparian zones), native plants with conservative traits (lower RSR or SRL) exhibit weak competitive ability. Concurrently, global nitrogen deposition favors fast-growing invasive species [95], while extended growing seasons under climate warming further amplify the competitive advantage of invasive plants phenologically similar to *S. canadensis* [96]. Invasive plants are more likely to successfully invade these native communities with weak competitive ability. Under global change scenarios, enhancing biotic resistance through trait-based selection and sowing of invasion-suppressing native plants can effectively restore environmental filtering capacity to constrain invader establishment.

## 4. Materials and Methods

### 4.1. Plant Material

Seeds of native plants and *S. canadensis* were collected from the wetland ecosystem of Hangzhou Bay in China (121°09′58″ E, 30°19′29″ N), where the invasion of *S. canadensis* is particularly severe. All collected native plant were tested for germination, and the species capable of germinating and growing normally at 25 °C were selected for further study (sprouting rate not less than 30%). The 32 selected species are listed in Table 3.

### 4.2. Experimental Design

The seeds collected from various plant species were decontaminated. Thirty seeds of each plant species were placed in culture dishes lined with filter paper and soaked in water. The germination rate of the seeds was tested in an environment maintained at 25 ± 1 °C, with the number of germinated seeds observed and recorded daily. Species with a germination rate exceeding 70% were chosen for pot experiments in a greenhouse (with flowerpots having a volume of 1 L). The experimental used an additive design [97] encompassing two treatment groups: a no-competition group and a competition group. Seeds were sown uniformly at the center of the flowerpots, followed by a regimen of scheduled daily irrigation. The seedlings in the flowerpots were gradually removed to ensure that each flowerpot in the no-competition group contained only one seedling after germination within 7 to 10 d, while each flowerpot in the competition group accommodated one native plant and one *S. canadensis* seedling. Each treatment was subjected to five replications. The study was conducted from March to June 2023 (the experiment lasted 70 days) at the experimental base of the Subtropical Forestry Research Institute of the Chinese Academy of Forestry (Hangzhou City, 119°57′10″ E, 30°3′35″ N). Hangzhou has a subtropical monsoon climate with red soil, an annual average temperature of 23 °C, a maximum temperature of 39 °C, a minimum temperature of −7 °C, and an annual average precipitation of 1299 mm. The soil type is red soil and test site was flat, with relatively uniform temperature and light conditions, and good air circulation.

### 4.3. Measurements

The plant height was measured once every 10 days after sowing using measuring tape (with an accuracy of 1 cm). At the end of the experiment, a root analysis system (Regent Instruments Inc., Sainte-Foy, Quebec, Canada) was employed to scan and analyze the root length, root tip number (RT), average root diameter and root surface area of different native plant species competing with *S. canadensis*. Additionally, leaf area (LA) was measured by WinFOLIA Pro 2015a software (Regent Instruments Inc., Sainte-Foy, Quebec, Canada), and the plants were dried to constant weight to determine the biomass of roots, stems (M_stem_), and leaves (M_leaf_). The root–shoot ratio, specific root length, and specific leaf area (SLA) were calculated. The corrected index of relative competitive intensity (CRCI) [98] was utilized to assess the competitive effects and responses between species:

CRCI_ij_ = arcsin[(M_0i_ − M_ij_)/max(M_0i_, M_ij_)] (1)

Note: M_0i_ is the dry weight of species i when planted alone, and M_i_ is the dry weight of plant i when species i is planted in mixture with species j; max(M_0i_, M_ij_) represents the larger value between M_0i_ and M_ij_. A higher value of CRCI_ij_ indicates that species j has a stronger inhibitory ability on species i, species j has a stronger competitive effect, and species i incurs a higher cost of competing against species j, and species i is more sensitive to competition.

The relative growth rate (RGR) of native plants and *S. canadensis* was calculated using plant height [99]:

(2) $${\mathrm{RGR}}_{\mathrm{ij}}\;=\;({\mathrm{lnH}}_{70}\;-\;{\mathrm{lnH}}_{0})/\Delta t$$

Note: H_0_ and H_70_ are the plant heights at the beginning and end of the experiment, respectively, and Δt is the time between measurements. In order to avoid the issue of zero values in logarithmic transformations, all plant height data were standardized by adding 1 before applying the logarithmic transformation.

### 4.4. Statistical Analyses

The One-way ANOVA of IBM SPSS Statistics 20 (IBM Corp., Armonk, NY, USA) was used to conduct a comparative analysis of the CRCI and functional traits (total biomass, root biomass, leaf area, plant height, RGR and RSR), where CRCI was analyzed with 0 to refer to no competitive effect. Multiple comparisons were performed using the Tukey method, and a significant difference between groups was considered when *p* < 0.05. The significant difference in CRCI and Functional traits between native plant control and mixed treatments was tested by independent samples T-test, and the difference was significant when *p* < 0.05. The native plants were categorized into two types: annuals and perennials. The Pearson correlation coefficient was used to perform correlation analysis between the CRCI of *S. canadensis* and the functional traits of native plants, as well as the correlation between the CRCI of different types of native plants and the functional traits of *S. canadensis*. The results were visualized using Origin 2024b (OriginLab Corp., Northampton, MA, USA). The CRCI of *S. canadensis* or native plants was used as the independent variable, and the functional traits of native plants or *S. canadensis* were used as the dependent variable for stepwise regression analysis (regression mode: stepwise selection), and the independent variables were considered to have a significant impact on the dependent variable when *p* < 0.05.

## 5. Conclusions

Most of the native species studied vary in their competitive abilities and are more competitive than *S. canadensis* in the seedling stage. The interspecific variation in competitive effects of native plants on *S. canadensis* can be explained by variation in root traits and plant height. Higher root–shoot ratio of annual plant species and smaller root diameter of perennial plant species may be the key functional traits responsible for higher competitive abilities. This provides a basis for selecting highly competitive native plant to suppress *S. canadensis*. Restoration of *S. canadensis* invaded habitats in its seedling stage may achieve high efficacy with highly competitive native species. This study is limited to the seedling stage, and it remains unclear whether these competitive effects can persist in the later stage of restoration. Further studies with prolonged duration under different environmental conditions are indispensable and more instructive for realistic control of *S. canadensis*.

## Figures and Tables

**Table 1:** Correlation between CRCI and functional traits.

Functional Traits	CRCI-s		CRCI-n	
	Correlation with Functional Traits of Native Plants Species		Correlation with Functional Traits of *S. canadensis*	
	Annual	Perennial	Annual	Perennial
LA	0.320 *	0.298 *	0.143	0.184
RL	0.468 *	0.228	0.233 *	0.018
RSA	0.479 *	0.184	0.220 *	−0.024
RD	0.296 *	−0.168	0.032	−0.085
RT	0.435 *	0.297 *	0.272 *	0.007
M_root_	0.498 *	0.255	0.144	0.032
M_stem_	0.474 *	0.397 *	0.105	0.104
M_leaf_	0.355 *	0.314 *	0.141	0.089
M	0.492 *	0.387 *	0.144	0.088
M_ag_	0.487 *	0.397 *	0.139	0.105
H_10_	0.177	−0.094	0.028	−0.019
H_20_	0.147	0.494 *	0.034	0.028
H_30_	0.274 *	0.460 *	−0.041	−0.023
H_40_	0.365 *	0.510 *	0.123	−0.085
H_50_	0.420 *	0.389 *	0.115	−0.084
H_60_	0.397 *	0.546 *	0.07	0.155
H_70_	0.436 *	0.539 *	0.095	0.17
RSR	0.139	−0.027	0.088	−0.136
SLA	−0.048	−0.199	−0.028	0.305 *
SRL	0.015	−0.198	0.091	−0.053

* represents the significant difference between the CRCIs and functional traits (*p* < 0.05).

**Table 2:** Summary of stepwise regression analysis of CRCI with the functional traits.

Dependent Variable	Native Plant Type	Adjusted R^2^	*F*	*p*
CRCI of *S. canadensis*	Annual	0.38	13.142	*p* < 0.05
	Perennial	0.387	10.521	*p* < 0.05
CRCI of native plants	Annual	0.056	6.886	*p* < 0.05
	Perennial	0.062	4.582	*p <* 0.05

**Table 3:** Native plant species used in this experiment, as well as their life form and family affiliation.

Latin Name	Family	Life Form (Annual/Perennial)
*Achyranthes bidentata*	Amaranthaceae	Perennial
*Ageratum houstonianum*	Asteraceae	Annual
*Artemisia lactiflora*	Asteraceae	Perennial
*Artemisia lavandulifolia*	Asteraceae	Perennial
*Astragalus sinicus*	Fabaceae	Perennial
*Brassica juncea*	Brassicaceae	Annual
*Carpesium abrotanoides*	Asteraceae	Perennial
*Crotalaria pallida*	Fabaceae	Perennial
*Cynanchum hemsleyanum*	Apocynaceae	Perennial
*Festuca elata*	Poaceae	Perennial
*Glycine soja*	Fabaceae	Perennial
*Hemisteptia lyrata*	Asteraceae	Annual
*Imperata cylindrica*	Poaceae	Perennial
*Ipomoea nil*	Convolvulaceae	Annual
*Kummerowia striata*	Fabaceae	Perennial
*Lactuca indica*	Asteraceae	Annual
*Ludwigia prostrata*	Onagraceae	Perennial
*Melilotus albus*	Fabaceae	Perennial
*Miscanthus sacchariflorus*	Poaceae	Perennial
*Persicaria lapathifolia*	Polygonaceae	Annual
*Persicaria maculosa*	Polygonaceae	Annual
*Phragmites australis*	Poaceae	Perennial
*Physalis angulata*	Solanaceae	Annual
*Rottboellia cochinchinensis*	Poaceae	Annual
*Rudbeckia hirta*	Asteraceae	Annual
*Rumex japonicus*	Polygonaceae	Perennial
*Sesbania cannabina*	Fabaceae	Annual
*Setaria pumila*	Poaceae	Annual
*Setaria viridis*	Poaceae	Annual
*Trifolium repens*	Fabaceae	Perennial
*Vicia hirsuta*	Fabaceae	Perennial
*Youngia japonica*	Asteraceae	Annual

## Data Availability

The original contributions presented in this study are included in the article/Supplementary Material. Further inquiries can be directed to the corresponding author.

## Supplementary Materials

The following supporting information can be downloaded at: https://www.mdpi.com/article/10.3390/plants14172806/s1. Figure S1: RGR of native plants and *S. canadensis*, Native-alone and Native-with *S.canadensis* represent the RGR of native plants grown alone and competing with *S. canadensis*, and *S. canadensis*-with native represents the RGR of *S. canadensis* competing with native. Red * indicates a significant difference between Native-alone and Native-with *S. canadensis*, black * represents a significant difference between Native-with *S. canadensis* and *S. canadensis*-with native, *p* < 0.05. Table S1: Percentage reduction in biomass of *S. canadensis* and native plants after competition. Lowercase letters following the values represent significant difference between the percent reduction in biomass of *S. canadensis* mixed with different native plants, uppercase letters represent significant difference between the percent reduction in biomass of native plants mixed with *S. canadensis*, and * represents the significant difference between the the percent reduction in biomass of native plants and *S. canadensis* when they are mixed. Table S2: Plant height of *S. canadensis* and native plants after competition. Lowercase letters indicate significant differences between plant heights of *S. canadensis*, and uppercase letters indicate significant differences between plant heights of native plants in the same treatment (mixed with *S. canadensis* or control). Table S3: Leaf area of *S. canadensis* and native plants. Lowercase letters indicate significant differences between leaf area of *S. canadensis*, and uppercase letters indicate significant differences between leaf area of native plants in the same treatment (mixed with *S. canadensis* or control). Table S4: Total biomass of *S. canadensis* and native plants. Lowercase letters indicate significant differences between total biomass of *S. canadensis*, and uppercase letters indicate significant differences between total biomass of native plants in the same treatment (mixed with *S. canadensis* or control). Table S5: Root biomass of *S. canadensis* and native plants. Lowercase letters indicate significant differences between root biomass of *S. canadensis*, and uppercase letters indicate significant differences between root biomass of native plants in the same treatment (mixed with *S. canadensis* or control). Table S6: RSR of *S. canadensis* and native plants. Lowercase letters indicate significant differences between RSR of *S. canadensis*, and uppercase letters indicate significant differences between RSR of native plants in the same treatment (mixed with *S. canadensis* or control). Table S7: Functional traits of *S. canadensis*-alone. Table S8: ANOVA table for functional traits. Table S9: Parameters of stepwise regression model for RCI and functional traits.

## Abbreviations

The following abbreviations are used in this manuscript:

CRCI	Corrected index of relative competitive intensity
RL	Root length
RT	Root tip
RD	Root diameter
RSA	Root surface area
RSR	Root–shoot ratio
SRL	Specific root length
LA	Leaf area
M_root_	Biomass of root
M_stem_	Biomass of stem
M_leaf_	Biomass of leaf
H_10–70_	Plant height of 10–70 days
M	Total biomass
