# Supplementary material for: Functional Traits of Native Plant Species That Inhibit the Seedling Growth of the Exotic Invader Solidago canadensis
Source: Plants (Basel). 2025 Sep 8;14(17):2806. doi: 10.3390/plants14172806 (PMC12430648; doi:10.3390/plants14172806)
Supplement: Supplementary file 1 [file plants-14-02806-s001.zip › plants-3706639-supplementary.pdf]

Supplementary Materials:

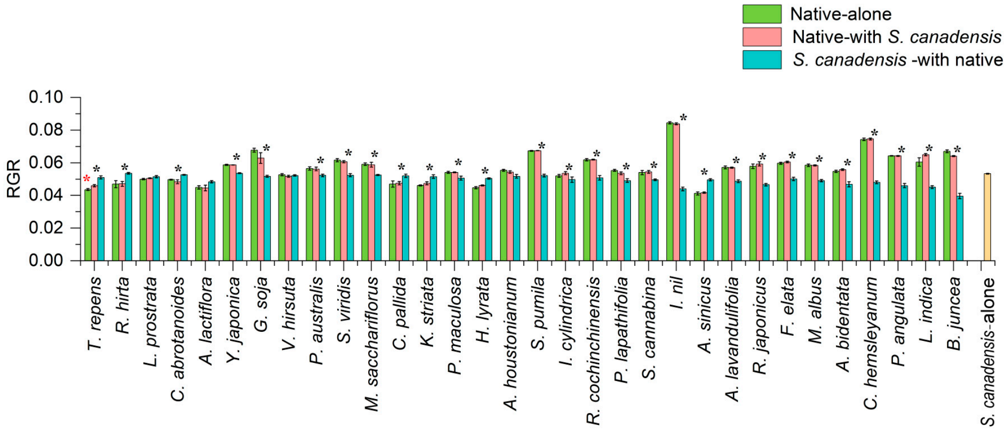

Figure S1. RGR of native plants and *S. canadensis*, Native-alone and Native-with *S.canadensis* represent the RGR of native plants grown alone and competing with *S. canadensis*, and *S. canadensis*-with native represents the RGR of *S. canadensis* competing with native. Red * indicates a significant difference between Native-alone and Native-with *S. canadensis*, black * represents a significant difference between Native-with *S. canadensis* and *S. canadensis*-with native, $p < 0.05$.

Table S1. Percentage reduction in biomass of *S. canadensis* and native plants after competition. Lowercase letters following the values represent significant difference between the percent reduction in biomass of *S. canadensis* mixed with different native plants, uppercase letters represent significant difference between the percent reduction in biomass of native plants mixed with *S. canadensis*, and * represents the significant difference between the the percent reduction in biomass of native plants and *S. canadensis* when they are mixed.

Names of native plants	Rate of biomass reduction (%)	
	S. canadensis	Native plants
T. repens	12±11 g	-50±34 B
R. hirta	24±12 fg	62±11 A
L. prostrata	42±6efg	31±08 AB
C. abrotanoides	43±7 defg	33±08 AB
A. lactiflora	48±7 cdefg	-48±18 B*
Y. japonica	49±14 cdefg	22±15 AB
G. soja	52±5 bcdef	30±18 AB
V. hirsuta	53±3 bcdef	23±21 AB
P. australis	59±5 abcdef	8±17 AB*
S. viridis	61±10 abcdef	1±07 AB*
M. sacchariflorus	62±2 abcde	-39±32 AB*
C. pallida	63±7 abcde	30±25 AB
K. striata	64±6 abcde	12±33 AB
P. maculosa	64±7 abcde	33±06 AB*
H. lyrata	64±8 abcde	51±11 AB
A. houstonianum	66±6 abcde	6±16 AB*
S. pumila	69±5 abcde	-2±12 AB*
I. cylindrica	70±8 abcde	-12±10 AB*
R. cochinchinensis	74±7 abcde	-14±20 AB*

P. lapathifolia	76±10 abcde	-13±32 AB*
S. cannabina	78±2 abcde	06±16 AB*
I. nil	78±7 abcde	14±17 AB*
A. sinicus	79±3 abcd	13±15 AB*
A. lavandulifolia	80±5 abc	-2±12 AB*
R. japonicus	80±4 abc	18±12 AB*
F. elata	82±5 abc	13±13 AB*
M. albus	84±2 abc	9±11 AB*
A. bidentata	85±5 abc	18±11 AB*
C. hemsleyanum	86±4 ab	-28±16 AB*
P. angulata	90±2 a	06±20 AB*
L. indica	93±1 a	-44±29 B*
B. juncea	93±2 a	26±12 AB*

Table S2. Plant height of *S. canadensis* and native plants after competition. Lowercase letters indicate significant differences between plant heights of *S. canadensis*, and uppercase letters indicate significant differences between plant heights of native plants in the same treatment (mixed with *S. canadensis* or control).

	Plant height (cm)		
**Names of native plants**	***S. canadensis*-with native plant**	**native plant-with *S. canadensis***	**native plant-alone**
T. repens	34.80±2.18 abcdef	24.40±1.24 MNO	20.20±0.86 MN
R. hirta	41.60±1.57 a	26.1±3.12 LMNO	27.10±4.22 JKLMN
L. prostrata	35.60±1.81 abcde	33.40±0.58 KLMNO	32.50±1.16 IJKLMN
C. abrotanoides	38.80±0.58 abc	29.30±2.35 LMNO	31.30±0.60 IJKLMN
A. lactiflora	28.80±1.46 cdefgh	22.50±2.64 NO	22.60±1.63 MN
Y. japonica	41.75±0.49 a	59.80±0.49 GHIJ	60.40±1.52 EFGHIJK
G. soja	36.40±1.44 abcd	88.00±14.90 CDEF	94.00±22.37 CDE
V. hirsuta	37.60±1.12 abc	36.50±1.63 JKLMNO	38.60±1.98 HIJKLMN
P. australis	38.20±2.31 abc	50.80±3.62 GHIJKL	51.60±3.41 GHIJKLMN
S. viridis	38.40±2.44 abc	68.10±3.51 DEFGH	74.50±4.37 DEFG
M. sacchariflorus	38.40±0.98 abc	61.90±5.70 FGHIJ	62.70±3.49 EFGHIJ
C. pallida	37.60±2.84 abc	27.70±2.13 LMNO	26.10±3.06 KLMN
K. striata	36.20±2.56 abcde	27.30±2.10 LMNO	24.10±0.66 LMN
P. maculosa	34.00±3.02 abcdef	43.0±0.86 HIJKLMNO	43.80±1.77 GHIJKLMN
H. lyrata	33.20±0.92 abcdefg	24.40±0.58 MNO	22.50±1.16 MN
A. houstonianum	36.80±2.89 abcd	44.70±2.65 HIJKLMN	47.60±2.06 GHIJKLMN
S. pumila	38.00±2.51 abc	111.70±1.00 C	110.00±2.03 C
I. cylindrica	32.20±3.57 abcdefg	42.50±2.53 IJKLMNO	37.50±1.99 HIJKLMN
R. cochinchinensis	34.60±3.23 abcdef	75.50±0.81 DEFG	75.80±3.54 CDEFG
P. lapathifolia	30.40±2.16 bcdefgh	41.50±2.60 IJKLMNO	47.60±1.88 GHIJKLMN
S. cannabina	31.20±1.07 abcdefgh	44.90±2.44 HIJKLMN	43.40±3.68 GHIJKLMN
I. nil	21.00±1.73 hi	352.10±14.71 A	370.00±17.82 A
A. sinicus	31.20±1.77 abcdefgh	17.80±0.68 O	17.60±1.22 N
A. lavandulifolia	29.50±1.69 cdefgh	46.50±6.71 HIJKLMN	54.70±3.75 FGHIJKLM
R. japonicus	25.20±1.39 efghi	63.70±6.09 EFGHI	57.00±6.14 FFGHIJKL

F. elata	32.80±2.78 abcdefg	67.10±2.18 DEFGHI	64.10±2.58 EFGHI
M. albus	30.20±1.59 bcdefgh	58.20±1.33 GHIJK	59.30±3.28 FGHIJK
A. bidentata	26.20±3.20 defghi	49.40±1.82 GHIJKLM	45.80±2.27 GHIJKLMN
C. hemsleyanum	28.00±1.92 cdefgh	185.9±8.46 B	181.40±10.91 B
P. angulata	24.40±2.11 fghi	88.20±1.91 CDE	88.9±1.44 CDEF
L. indica	22.60±1.40 ghi	93.80±4.48 CD	72.40±11.67 EFGH
B. juncea	15.40±1.72 i	87.40±2.50 CDEF	108.50±6.14 CD

Table S3. Leaf area of *S. canadensis* and native plants. Lowercase letters indicate significant differences between leaf area of *S. canadensis*, and uppercase letters indicate significant differences between leaf area of native plants in the same treatment (mixed with *S. canadensis* or control).

Names of native plants	Leaf area (cm^2)		
	***S. canadensis*-with native plant**	**native plant-with *S. canadensis***	**native plant-alone**
P. australis	354.52±20.96 bcdef	47.48±2.39 IJ	55.65±10.35 KL
C. abrotanoides	546.37±39.54 ab	917.13±137.47 BCDEFG	1241.04±99.44 BCDE
A. lactiflora	381.84±41.56 bcde	838.92±120.41 BCDEFGH	454.91±42.80 GHIJKL
B. juncea	73.08±27.91 f	211.17±73.29 GHIJ	608.60±130.27 FGHIJKL
C. pallida	410.79±53.90 bcde	125.73±27.00 HIJ	165.92±28.65 IJKL
Y. japonica	480.47±85.01 abcd	267.45±30.84 GHIJ	284.24±132.02 HIJKL
H. lyrata	299.98±59.98 bcdef	485.70±155.72 EFGHIJ	680.93±146.37 DEFGHIJ
M. sacchariflorus	411.16±29.85 bcde	68.05±11.95 IJ	54.41±13.87 KL
C. hemsleyanum	118.83±31.02 ef	1821.01±263.65 A	1498.29±75.34 ABC
S. cannabina	266.54±20.09 bcdef	200.08±66.66 GHIJ	143.59±37.27 IJKL
P. maculosa	322.17±57.02 bcdef	1067.40±76.43 BCDEF	1176.43±131.56 CDEF
G. soja	343.47±60.64 bcdef	204.63±62.28 GHIJ	304.17±76.69 HIJKL
L. prostrata	478.48±84.34 abcd	191.81±18.63 GHIJ	289.44±19.72 HIJKL
A. sinicus	234.78±41.48 cdef	561.28±59.76 DEFGHIJ	643.58±63.90 EFGHIJK
S. pumila	226.84±34.09 def	1181.35±206.32 ABCDE	1275.69±202.14 BCD
I. cylindrica	304.65±61.31 bcdef	24.67±3.30 J	24.49±2.23 L
R. cochinchinensis	213.84±48.58 def	101.55±15.19 HIJ	78.17±6.00 JKL
P. angulata	115.48±23.46 ef	1917.59±427.42 A	2105.06±153.62 A
F. elata	187.85±53.32 def	525.98±41.36 DEFGHIJ	578.77±108.17 FGHIJKL
A. houstonianum	301.76±47.28 bcdef	1018.76±159.29 BCDEF	1111.45±110.62 CDEF
L. indica	70.51±5.28 f	1267.54±336.15 ABCD	809.17±344.35 DEFGH
A. bidentata	147.79±53.37 ef	1500.52±145.32 AB	1802.62±115.74 AB
M. albus	191.96±15.69 def	237.28±26.88 GHIJ	268.44±46.90 HIJKL
P. lapathifolia	223.60±76.97 def	794.27±96.73 BCDEFGHI	753.69±50.10 DEFGHI
I. nil	72.09±12.61 f	648.09±118.01 CDEFGHIJ	675.00±72.35 DEFGHIJ
R. japonicus	196.39±37.36 def	368.68±31.01 FGHIJ	581.30±48.73 FGHIJKL
K. striata	363.24±50.28 bcdef	34.43±7.03 J	49.00±6.37 KL
V. hirsuta	526.80±49.43 abc	42.75±9.44 J	50.72±4.74 KL
S. viridis	289.41±55.51 bcdef	1321.85±147.15 ABC	1488.63±50.60 BC
T. repens	760.33±85.76 a	90.12±16.87 HIJ	68.71±11.03 JKL
R. hirta	529.99±108.64 abc	234.14±49.31 GHIJ	819.07±48.53 DEFGH
A. lavandulifolia	166.90±33.92 ef	1041.44±113.01 BCDEF	1044.14±252.52 CDEFG

Table S4. Total biomass of *S. canadensis* and native plants. Lowercase letters indicate significant differences between total biomass of *S. canadensis*, and uppercase letters indicate significant differences between total biomass of native plants in the same treatment (mixed with *S. canadensis* or control).

Names of native plants	Total biomass (g)		
	S. canadensis-with native plant	native plant-with *S. canadensis*	native plant-alone
P. australis	1.53±0.18 cdefg	0.18±0.01 G	0.23±0.04 J
C. abrotanoides	2.11±0.25 bcd	0.25±0.75 DEFG	5.71±0.65 CDEFG
A. lactiflora	1.91±0.27 bcde	0.27±0.47 CDEF	3.32±0.14 FGHIJK
B. juncea	0.25±0.07 g	0.07±0.98 BCDE	7.66±0.96 BCDE
C. pallida	1.36±0.27 defg	0.27±0.11 G	0.53±0.04 J
Y. japonica	1.88±0.53 bcde	0.53±0.15 FG	1.63±0.23 HIJK
H. lyrata	1.11±0.25 defg	0.25±0.88 EFG	3.21±1.10 GHIJK
M. sacchariflorus	1.42±0.09 cdefg	0.09±0.08 G	0.33±0.03 J
C. hemsleyanum	0.52±0.14 efg	0.14±1.22 ABC	7.13±0.62 BCDE
S. cannabina	0.82±0.06 defg	0.06±0.19 EFG	1.74±0.23 HIJK
P. maculosa	1.34±0.26 defg	0.26±1.07 ABCD	8.51±1.44 BC
G. soja	1.68±0.21 cdef	0.21±0.28 FG	1.62±0.13 HIJK
L. prostrata	2.16±0.22 bcd	0.22±0.05 FG	1.00±0.10 IJK
A. sinicus	0.77±0.12 defg	0.12±0.10 FG	1.47±0.26 HIJK
S. pumila	1.15±0.19 defg	0.19±1.85 A	9.96±1.07 AB
I. cylindrica	1.10±0.28 defg	0.28±0.01 G	0.10±0.01 J
R. cochinchinensis	0.96±0.25 defg	0.25±0.10 FG	0.79±0.12 JK
P. angulata	0.36±0.09 fg	0.09±2.00 ABC	9.77±0.88 AB
F. elata	0.67±0.20 efg	0.20±0.30 EFG	2.57±0.30 GHIJK
A. houstonianum	1.28±0.24 defg	0.24±0.94 DEFG	4.84±0.53 DEFGH
L. indica	0.25±0.02 g	0.02±1.78 A	8.39±1.54 BCD
A. bidentata	0.56±0.20 efg	0.20±0.93 AB	12.28±0.63 A
M. albus	0.60±0.06 efg	0.06±0.11 EFG	1.61±0.22 HIJK
P. lapathifolia	0.90±0.36 defg	0.36±0.68 DEFG	4.42±0.59 EFGHIJ
I. nil	0.83±0.27 defg	0.27±0.70 DEFG	4.59±0.50 EFGHI
R. japonicus	0.73±0.16 defg	0.16±0.74 ABCD	8.71±1.05 ABC
K. striata	1.34±0.24 defg	0.24±0.03 G	0.18±0.02 J
V. hirsuta	1.76±0.11 cdef	0.11±0.06 G	0.26±0.03 J
S. viridis	1.46±0.38 cdefg	0.38±0.48 ABCD	6.97±0.33 BCDEF
T. repens	3.24±0.41 ab	0.41±0.09 FG	0.37±0.05 J
R. hirta	2.81±0.45 abc	0.45±0.31 FG	3.36±0.53 FGHIJK
A. lavandulifolia	0.75±0.17 defg	0.17±0.83 ABCD	8.71±1.30 ABC

Table S5. Root biomass of *S. canadensis* and native plants. Lowercase letters indicate significant differences between root biomass of *S. canadensis*, and uppercase letters indicate significant differences between root biomass of native plants in the same treatment (mixed with *S. canadensis* or control).

Names of native plants	Root biomass (g)

	S. canadensis-with native plant	native plant-with *S. canadensis*	native plant-alone
P. australis	0.17±0.03 cdefgh	0.03±0.00 H	0.02±0.00 F
C. abrotanoides	0.17±0.01 cdefgh	0.80±0.21 BCDEF	1.13±0.21 BCD
A. lactiflora	0.21±0.03 cdef	1.44±0.05 BCD	0.99±0.05 BCD
B. juncea	0.03±0.01 fgh	0.45±0.07 FGH	0.72±0.07 BCDEF
C. pallida	0.14±0.03 defgh	0.03±0.01 H	0.03±0.01 F
Y. japonica	0.21±0.08 bcde	0.05±0.01 H	0.08±0.01 F
H. lyrata	0.12±0.02 defgh	0.20±0.04 FGH	0.19±0.04 EF
M. sacchariflorus	0.13±0.01 defgh	0.07±0.01 GH	0.04±0.01 F
C. hemsleyanum	0.07±0.02 defgh	0.80±0.08 BCDEF	0.72±0.08 BCDEF
S. cannabina	0.09±0.01 defgh	0.19±0.04 FGH	0.13±0.04 F
P. maculosa	0.13±0.03 defgh	1.19±0.22 BCDE	1.27±0.22 BC
G. soja	0.20±0.03 cdefg	0.07±0.03 GH	0.11±0.03 F
L. prostrata	0.23±0.03 bcd	0.07±0.01 GH	0.12±0.01 F
A. sinicus	0.08±0.02 defgh	0.16±0.04 FGH	0.22±0.04 EF
S. pumila	0.12±0.03 defgh	1.49±0.11 BC	1.22±0.11 BC
I. cylindrica	0.11±0.03 defgh	0.01±0.00 H	0.02±0.00 F
R. cochinchinensis	0.12±0.03 defgh	0.21±0.02 FGH	0.15±0.02 EF
P. angulata	0.05±0.02 efgh	0.77±0.15 DEFG	0.91±0.15 BCDE
F. elata	0.05±0.01 efgh	0.38±0.06 FGH	0.44±0.06 DEF
A. houstonianum	0.10±0.02 defgh	0.54±0.12 EFGH	0.56±0.12 CDEF
L. indica	0.02±0.01 h	1.49±0.23 B	1.43±0.23 B
A. bidentata	0.05±0.03 defgh	2.42±0.22 A	2.74±0.22 A
M. albus	0.12±0.06 defgh	0.14±0.04 FGH	0.21±0.04 EF
P. lapathifolia	0.10±0.04 defgh	0.53±0.09 EFGH	0.61±0.09 CDEF
I. nil	0.03±0.01 gh	0.19±0.02 FGH	0.13±0.02 F
R. japonicus	0.10±0.02 defgh	2.50±0.53 A	3.33±0.53 A
K. striata	0.15±0.03 cdefgh	0.01±0.00 H	0.01±0.00 F
V. hirsuta	0.16±0.01 cdefgh	0.02±0.00 H	0.02±0.00 F
S. viridis	0.16±0.05 cdefgh	0.79±0.09 CDEF	0.72±0.09 BCDEF
T. repens	0.39±0.05 ab	0.05±0.00 H	0.02±0.00 F
R. hirta	0.33±0.07 abc	0.08±0.01 GH	0.23±0.01 EF
A. lavandulifolia	0.09±0.02 defgh	1.18±0.24 BCDE	1.28±0.24 BC

Table S6. RSR of *S. canadensis* and native plants. Lowercase letters indicate significant differences between RSR of *S. canadensis*, and uppercase letters indicate significant differences between RSR of native plants in the same treatment (mixed with *S. canadensis* or control).

Names of native plants	RSR		
	S. canadensis-with native plant	native plant-with *S. canadensis*	native plant-alone
P. australis	0.12±0.02 ab	0.18±0.04 CDEFG	0.08±0.02 DE
C. abrotanoides	0.09±0.01 ab	0.26±0.01 CDE	0.24±0.02 BCDE
A. lactiflora	0.12±0.01 ab	0.44±0.05 AB	0.43±0.03 AB
B. juncea	0.16±0.03 ab	0.09±0.01 EFG	0.11±0.01 CDE
C. pallida	0.11±0.01 ab	0.11±0.02 EFG	0.06±0.01 DE

Y. japonica	0.14±0.04 ab	0.05±0.01 G	0.05±0.00 DE
H. lyrata	0.11±0.02 ab	0.16±0.05 DEFG	0.14±0.03 CDE
M. sacchariflorus	0.10±0.01 ab	0.25±0.11 CDEF	0.12±0.03 CDE
C. hemsleyanum	0.15±0.03 ab	0.11±0.02 EFG	0.11±0.01 CDE
S. cannabina	0.13±0.01 ab	0.13±0.01 EFG	0.09±0.01 DE
P. maculosa	0.11±0.01 ab	0.20±0.01 CDEFG	0.19±0.03 BCDE
G. soja	0.13±0.02 ab	0.08±0.01 G	0.24±0.16 BCDE
L. prostrata	0.12±0.01 ab	0.11±0.02 EFG	0.14±0.01 CDE
A. sinicus	0.11±0.02 ab	0.18±0.05 DEFG	0.19±0.03 BCDE
S. pumila	0.11±0.01 ab	0.17±0.01 DEFG	0.14±0.01 CDE
I. cylindrica	0.10±0.01 ab	0.16±0.02 DEFG	0.19±0.02 BCDE
R. cochinchinensis	0.15±0.02 ab	0.35±0.04 BC	0.25±0.02 BCDE
P. angulata	0.14±0.03 ab	0.09±0.00 EFG	0.10±0.01 CDE
F. elata	0.09±0.02 b	0.21±0.03 CDEFG	0.21±0.03 BCDE
A. houstonianum	0.08±0.02 b	0.14±0.02 EFG	0.13±0.02 CDE
L. indica	0.10±0.02 ab	0.16±0.01 DEFG	0.34±0.16 BC
A. bidentata	0.09±0.02 ab	0.33±0.03 BCD	0.29±0.01 BCD
M. albus	0.30±0.18 a	0.11±0.01 EFG	0.14±0.01 CDE
P. lapathifolia	0.11±0.03 ab	0.14±0.02 EFG	0.16±0.01 CDE
I. nil	0.06±0.03 b	0.05±0.01 G	0.03±0.00 E
R. japonicus	0.21±0.08 ab	0.60±0.06 A	0.61±0.05 A
K. striata	0.13±0.01 ab	0.08±0.01 FG	0.06±0.01 DE
V. hirsuta	0.10±0.01 ab	0.10±0.02 EFG	0.06±0.01 DE
S. viridis	0.11±0.01 ab	0.13±0.02 EFG	0.11±0.01 CDE
T. repens	0.14±0.01 ab	0.10±0.03 EFG	0.05±0.01 DE
R. hirta	0.13±0.01 ab	0.08±0.02 FG	0.08±0.01 DE
A. lavandulifolia	0.13±0.01 ab	0.19±0.02 CDEFG	0.18±0.02 CDE

Table S7. Functional traits of *S. canadensis*-alone

**Functional traits**	***S. canadensis*-alone**		**unit**
	**Mean**	**SE**	
Plant height	40.90	0.80	cm
Leaf area	750.11	55.65	cm^2
Total biomass	3.70	0.28	g
Root biomass	0.43	0.02	g
RSR	0.14	0.01	-

Table S8. ANOVA table for functional traits.

**Functional traits**	***S. canadensis*-with native plant**			**native plant-with *S. canadensis***			**native plant-alone**		
	**df**	***F***	***p***	**df**	***F***	***p***	**df**	***F***	***p***
Plant height	32	9.872	< 0.05	31	163.994	< 0.05	31	106.465	< 0.05
Total biomass	32	12.377	< 0.05	31	19.195	< 0.05	31	29.938	< 0.05

Leaf area	32	12.434	< 0.05	31	16.298	< 0.05	31	25.158	< 0.05
Root biomass	32	10.715	< 0.05	31	28.463	< 0.05	31	31.396	< 0.05
RSR	32	1.134	< 0.05	31	14.002	< 0.05	31	7.253	< 0.05

Table S9. Parameters of stepwise regression model for RCI and functional traits.

Dependent variable	Narive plant type	Constant	Coefficients	p
RCI of *S. canadensis*	Annual	RT	-0.319	< 0.05
		M_{stem}	0.731	< 0.05
		H_{10}	0.265	< 0.05
		RSR	0.302	< 0.05
		SRL	0.379	< 0.05
	Perennial	RD	-0.297	< 0.05
		M_{root}	0.334	< 0.05
		H_{60}	0.52	< 0.05
RCI of native plant	Annual	RT	0.256	< 0.05
	Perennial	SLA	0.282	< 0.05
